# Sugammadex induced bradycardia and hypotension: A case report and literature review

**DOI:** 10.1097/MD.0000000000026796

**Published:** 2021-07-30

**Authors:** I-Chia Teng, Ying-Jen Chang, Yao-Tsung Lin, Chin-Chen Chu, Jen-Yin Chen, Zhi-Fu Wu

**Affiliations:** a Department of Anesthesiology, Chi Mei Medical Center, Tainan City, Taiwan; b Department of Food Science and Technology, Chia Nan University of Pharmacy and Science, Tainan, Taiwan; c Department of Anesthesiology, Kaohsiung Medical University Hospital, Kaohsiung Medical University, Kaohsiung, Taiwan; d Department of Anesthesiology, Tri-Service General Hospital and National Defense Medical Center, Taipei, Taiwan.

**Keywords:** bradycardia, cardiac arrest, hypotension, sugammadex

## Abstract

**Rationale:**

There is evidence that sugammadex can facilitate extubation post-surgery and attenuate postoperative pulmonary complications resulting from postoperative residual neuromuscular blockade. However, it may induce adverse effects, including bronchospasm, laryngospasm, bradycardia, hypotension, and cardiac arrest. Here, we present a case of sugammadex-induced bradycardia and hypotension.

**Patient concerns:**

An 82-year-old female received video-assisted thoracic surgery decortication and wedge resection of the lung for empyema. Post-surgery, she developed bradycardia, hypotension, hypoxia, and weakness.

**Diagnoses:**

The patient was suspected to have sugammadex-induced bradycardia, hypotension, hypoxia and weakness.

**Interventions:**

The patient received immediate treatment with atropine (0.5 mg) for bradycardia. Glycopyrrolate (0.1 mg) and neostigmine (1 mg) were administered to improve the train-of-four (TOF) ratio.

**Outcomes:**

Following initial management, we observed improvement in the hemodynamics of the patient. She was discharged without any sequelae.

**Lessons:**

Sugammadex-induced bradycardia or cardiac arrest are rare; however, anesthesiologists must consider the possibility of the occurrence of such events and initiate appropriate management measures. Immediate treatment with atropine and inotropic or vasopressors is warranted if the patient presents with bradycardia.

## 1 Introduction

Neuromuscular blocking agents are often employed to facilitate intubation, mechanical ventilation, and favorable surgical conditions. However, postoperative pulmonary complications, such as pulmonary hemorrhage, difficulty breathing, reintubation, and prolongation of the patient's length of stay could be attributed to postoperative residual neuromuscular blockade.^[1,2]^

Reversal agents are used to speed up recovery time from neuromuscular blockade and prevent postoperative residual neuromuscular blockade.^[3]^ In contrast to acetylcholinesterase inhibitors, sugammadex has been demonstrated to shorten extubation time, resulting in improved operating room turnover in clinical anesthesia settings and attenuation of postoperative pulmonary complications.^[4,5]^ However, sugammadex has been associated with several adverse effects, including bronchospasm,^[6]^ pulmonary edema,^[6]^ desaturation, hypotension, laryngospasm,^[7]^ bradycardia, and cardiac arrest.^[8–16]^ Here, we present a case of sugammadex-induced bradycardia, hypotension, and hypoxia in a patient following video-assisted thoracic surgery decortication and wedge resection of the lung. In addition, we analyzed adult cases of bradycardia or hypotension following sugammadex administration between January 2014 and January 2021 with a detailed literature review as well.

## 2 Case presentation

The patient was an 82-year-old woman (146 cm, 44 kg) diagnosed with hypertensive heart disease with no known history of allergy. Upon being diagnosed with empyema, she was scheduled for video-assisted thoracic surgery decortication and wedge resection of the lung. Pre-procedural electrocardiography revealed a normal sinus rhythm. Cardiac echocardiography showed a left ventricular ejection fraction of 71% and impaired left ventricular relaxation. Abnormal laboratory data demonstrated a white blood cell count of 15500/uL and C-reactive protein level of 220.7 mg/L. Vital signs were unremarkable except for oxygen saturation being around 90% to 95% on room air.

Routine monitoring included electrocardiography (lead II), noninvasive blood pressure, pulse oximetry, end-tidal carbon dioxide measurement, entropy, and the use of neuromuscular transmission monitors (NMTM). General anesthesia was induced with thiamylal (225 mg), rocuronium (50 mg), lidocaine (40 mg), fentanyl (75 mg), and glycopyrrolate (0.1 mg). The patient was intubated with a 32 Fr left double-lumen tube, and inspected using a fiberoptic bronchoscope. Following tracheal intubation, a right radial arterial line and central venous catheter were inserted. Anesthesia was maintained with sevoflurane (0.5–0.9), minimum alveolar concentration and propofol (20 mL/h) ti4trated to effect under entropy. In addition, the patient received a continuous dose of rocuronium (15–10 mg/h) under NMTM. Ventilation was adjusted to maintain an end-tidal carbon dioxide of 35 to 45 mmHg. The surgery was completed uneventfully in 2 hours and 40 minutes. At the completion of the procedure, arterial blood pressure of the patient was estimated to be 124/85 mmHg; heart rate, 65 beats per minute (bpm); SpO_2,_ 99%; NMTM count, 4; and train-of-four (TOF) ratio, 64. The patient regained consciousness and spontaneous breathing. A dose of 200 mg sugammadex was next administered. One minute following sugammadex administration, the patient developed sinus bradycardia with 34 bpm without diffuse ST depression, hypotension (67/34 mmHg), hypoxia (SpO_2_: 65%), with an NMTM count of 0, and TOF ratio of 0. The patient was immediately administered atropine (0.5 mg) intravenously, following which her hemodynamics improved, with a corresponding heart rate of 65 bpm, SBP of 89/50 mmHg, and SpO_2_ of 93%. Subsequent administration of glycopyrrolate (0.1 mg) and neostigmine (1 mg) improved the TOF ratio to 69. Arterial blood gas values were as follows: pH, 7.291; PaO_2_, 75 mmHg; PaCO_2_, 51.4 mmHg; HCO_3_^−^, 22.8 mmol/L; lactate, 1.1 mmol/L; hemoglobin, 12.6 g/dL; and hematocrit, 36.7%. The anesthesiologist replaced the double-lumen tube with a single-lumen 4endotracheal tube, following which the patient was transferred to the intensive care unit for further care. The patient was extubated and discharged 4 and 10 days post-surgery, respectively, without any sequelae.

## 3 Discussion

PubMed and Cochrane Database were searched for the terms “sugammadex AND (bradycardia operating room cardiac arrest)” in January 2021. Articles between January 2014 and January 2021 were included regardless of the type of publication or journal. The full text of the articles was retrieved. The authors assessed the articles using the following set of criteria:

1. the article is written in English;
2. the study is a case report or case series;
3. the study includes a description of the dose of sugammadex used, its adverse effects, and the treatment strategy;
4. the subjects are adult patients.

Information such as the type of publication (case report), year of publication, description of adverse effects, treatment administered for the reaction, and patient outcome were extracted from the articles. The results have been summarized in Table 1.

**Table 1:** Literature review of reported cases of sugammadex induced bradycardia or cardiac arrest.

	Patient Information				
Authors	Dose of Sugammadex	Signs and symptoms	Management	Outcome	Mechanism
Obara et al^[12]^	73 y/oMale77 kg, 178 cmSugammadex 200 mg (2.60 mg/kg)	• Hypotension: SBP unmeasurable (6 min after sugammadex)• ECG: ST depression, polymorphic ventricular premature contraction, and then cardiac arrest• Unconscious• Flushed on entire body trunk and lower limbs	• Fluid resuscitation• Phenylephrine 0.1 mg for 2 times• Epinephrine 0.1 mg• Suspecting primary cardiac ischemia, lidocaine (100 mg over 2 min) and nicorandil (2 mg/h) were also administered to prevent further progression of cardiac ischemia and arrhythmia• Re-intubation• CPR 10 min: epinephrine 3 mg i.v., defibrillations 2 times for VF• Suspecting an allergic reaction, methylprednisolone 1 g, hydroxyzine hydrochloride 50 mg, and a large volume of fluids	• SBP 70 mmHg, sinus heart rhythm 110 bpm• ICU• Consciousness recover 1 hr. after shock• The serum tryptase level 3 h. after the event was elevated to 9.6 μg/L (normal range, 1.2–5.7)• Skin prick test: positive reaction of sugammadex	Anaphylaxis
Bhavani et al^[8]^	41 y/oMale72 kgSugammadex 300 mg (4.17 mg/kg)	**Case 1**• Bradycardia (HR: 25 bpm) (2 min after sugammadex)• No palpable peripheral pulses	• CPR• Epinephrine 1 mg i.v.• Re-intubation• Ventilation with 100% O_2_	• Spontaneous cardiac activity• ICU• Tryptase concentration drawn shortly after resuscitation: 1.7 mg litre1 (normal < 11)• Cardiac enzymes, troponins: normal• ECG: no evidence of ischemia• Die on 15th day	Unknown
	60 y/oFemale88 kgSugammadex 200 mg (2.27 mg/kg)	**Case 2**• Bradycardia (HR: 30 bpm) (1 min after sugammadex)• Asystole• Hypotension• No palpable pulse	• CPR• Epinephrine 30 mg i.v.	• Spontaneous circulation and normal hemodynamics at the end of the 5th cycle CPR• Re-intubated during CPR• Extubated 15 min later• Discharged 48 hrs. later	Unknown
Sanoja and Toth^[14]^	60 y/oMale82 kgBMI 28.4Sugammadex 200 mg (2.4 mg/kg)	• Bradycardia (HR: 35 bpm) (< 1 mins after sugammadex)• MAP dropped to mid-30s• No rash or urticaria• Bilateral breath sounds clear• Peak airway pressure remained 18 cmH_2_O; end-tidal CO_2_: 38 mmHg• End-tidal CO_2_ fell to 10 mm Hg• Carotid pulses (-)• PEA	• Atropine 1 mg (no effect)• CPR• Epinephrine 1 mg every 3 minutes for a total of 7 mg• Calcium chloride 1 gm until return of spontaneous circulation	• Spontaneous circulation, with HR > 100 bpm and MAP > 90 mm Hg• ICU• Transthoracic echocardiogram, electrocardiogram, chest X-ray, computed tomography chest, urine toxicology: all unremarkable• Tryptase level: not obtained (owing to a lack of signs or symptoms of allergic reaction)• Discharged on day 7 with no discernible sequelae	Unknown
Yanai and Ariyos^[15]^	71 y/oFemale65 kgSugammadex 200 mg (3.08 mg/kg)	Episode 1• Desaturation (2 min after sugammadex)• VF• No rash on the skin	• Mask ventilation• CPR and defibrillation• Re-intubated 18 mins after resuscitation	• Unconscious (GCS: E1VTM1) without sedation• BP 100/60 mmHg, HR 100 bpm• RR 20 beats/min with spontaneous breathing• SpO_2_ level was 100% while breathing 100% oxygen• 12-lead ECG: sinus tachycardia without specific ST segment elevation or depression• TTE: normal EF with no regional wall motion abnormalities• Blood tests, chest X-ray, brain CT scan, contrast-enhanced CT scans of chest and abdomen → no remarkable findings• Troponin I level 1 hr. after cardiac arrest: 0.219 ng/mL (ref: 0–0.028 ng/mL)• ICU, discharged from ICU on day 14• Coronary CT angiography on day 45 → no marked coronary artery disease• Transferred to a rehabilitation hospital on day 61	Kounis Syndrome (Anaphylaxis)
	71 y/oFemale65 kgSugammadex 130 mg (2 mg/kg)	Episode 2• Blood pressure drop suddenly• Bradycardia (without mention of HR)• PEA4	• Noradrenaline i.v. drip	• Spontaneous circulation after 13 min of resuscitation• ECG: diffuse ST depression• TTE: diffused, severely depressed left ventricular wall motion• Coronary angiogram: multiple spasms in RCA, resolved via intracoronary administration of nitroglycerin• Troponin I: 0.245 ng/mL• At 72 mins, tryptase level 81.2 μg/L (ref: 1.2–5.7 μg/mL)• ICU: 40 mg methylprednisolone i.v. and continuous infusion of nicorandil• Recovered by day 3 and so was extubated• Discharged from ICU on day 5• Skin prick test: positive reaction to sugammadex• Discharged to a rehabilitation hospital on day 26	Kounis Syndrome (Anaphylaxis)
Yoshida et al^[16]^	50 y/oFemale79.2 kg, 156 cmSugammadex 200 mg (2.53 mg/kg)	• Bradycardia (HR from 87 bpm to 36 bpm) (1 min after sugammadex)• Hypotension (41/20 mmHg)• ST depression in lead II• Lack of signs suggesting allergic reactions, such as skin rash or urticaria	• Atropine 0.5 mg i.v. (hemodynamics did not improve)• Adrenaline 0.5 mg i.v. 2 min after atropine• Trachea intubated	• ICU (approximately 1 hr. after bradycardia occurred)• HR 130 bpm and BP 100/54 mmHg• Spontaneous breathing with low tidal volume leading to hypercapnia (end-tidal CO_2_ 58 mmHg) and alveolar hypoventilation (SpO_2_ 93% [FiO_2_ 1.0])• Bilateral breath sounds clear• Normal systolic function of both ventricles• BIS: 70–80• ECG: down sloping ST depression in leads II, III, aVF, and V3–6, as well as ST elevation in lead aVR, were noted, MI was considered• Serum tryptase, histamine were not assessed• Extubated 9 hrs after admitted to ICU• Discharged on POD 8	Unknown
Mirza et al^[11]^	82 y/oMale68.97 kgSugammadex 200 mg (2.9 mg/kg)	• Bradycardia (without mention of HR)• Asystole• PEA• Ventricular rhythms	• Glycopyrrolate 0.2 mg• Ephedrine 10 mg• Multiple doses of epinephrine• CPR• Defibrillation• Norepinephrine infusion	• Death	Unknown
Carmen et al^[10]^	80 y/oMale55 kg, 158 cmSugammadex 200 mg (3.64mg/kg)	• Severe bradycardia (HR < 35 bpm) (1 minute after sugammadex)• SBP < 50 mmHg• Asystole and cardiac arrest	• Total of 10 mg ephedrine i.v.• Atropine 1 mg i.v.• CPR for 1 min, restoring spontaneous cardiac activity	• ICU• Cardiac enzymes and troponins: normal• ECG and transthoracic echocardiography: not show any pathological sign• Spontaneous respiration during the following 3 days in ICU• Discharged uneventfully on postoperative day 10	Unknown
Murat Bilgi et al^[9]^	56y/oMale77 kg, 163 cmSugammadex 200 mg (2.6 mg/kg)	• Bradycardia (HR 35 bpm) (2 min after sugammadex)• BP: 124/81 mmHg• SpO_2_ 99%, airway pressure 20 cmH_2_O, end tidal CO_2_ 42 mmHg• After 0.5 mg i.v. atropine, HR increased to 55 bpm, then decreased to 30–35 bpm again	• Atropine 0.5 mg i.v.• Total dose of 2 mg atropine	• HR 63 bpm• Adequate spontaneous respiration• Vital findings stable for 1 hr• Discharged	Unknown
Evangelia Samara et al^[13]^	54y/oMale175 cm, 75 kgSugammadex 200 mg (2.7 mg/kg)	• Unresponsive• Apnea• Pulseless• Asystole• SpO_2_ 45%	• CPR (after 5 mins of CPR, EtCO_2_: 15 mmHg)• Epinephrine 8 mg• Amiodarone 450 mg• Defibrillated 6 times• Re-intubation	• Sinus rhythm (after 40 mins)• Discharged after 2 more days	Unknown
Bedirli et al^[17]^	22y/oFemale213 cm, 85 kgSugammadex 340 mg (4 mg/kg)	• Bradycardia (HR 43 bpm) (Immediately after sugammadex administration)• Hypotension (43/25 mmHg)• Arrhythmia: PVC and bigeminy with the heart rate of 125 beats/min occurred• Face and upper body were flushed• Airway pressure: 40 cmH_2_O• Bronchospasm was diagnosed by wheezing• Peripheral oxygen saturation: 86%	• Ephedrine 10 mg i.v.• Rapid infusion of lactated Ringer's solution• Epinephrine 50-μg i.v.• Lidocaine 60 mg i.v.• Epinephrine 50-μg i.v. was repeated• Methylprednisolone 125 mg i.v.• Famotidine 20 mg i.v.• Pheniramine maleat i.v.• Continuous infusion of norepinephrine 0.04 μg/kg/min for 12 h	• BP: 90/50 mm Hg• Extubated (3 hrs after sugammadex administration)• ICU• Serum Ig E, tryptase levels assessed 3 and 24 hrs after the onset of anaphylactic reaction: within normal range• Discharged to home with normal ECG	Anaphylaxis
Our case	82y/oFemale 146 cm, 44 kgSugammadex 200 mg (4.55 mg/kg)	• Bradycardia (34 bpm without diffuse ST depression) (1 min after sugammadex)• Hypotension (67/34 mmHg)• Hypoxia (SpO_2_: 65%)• NMTM count: 0• TOF ratio: 0	• Atropine 0.5 mg i.v.• Glycopyrrolate 0.1 mg and neostigmine 1 mg	• ICU• Extubated and discharged 4 and 10 d after surgery without any sequel	Unknown

We came across 11 cases of bradycardia and/or cardiac arrest.^[8–17]^ These cases were further categorized into cardiac events associated with bradycardia (9 out of 11),^[8–11,14–17]^ cardiac arrest/asystole (5 out of 11),^[8,10–13]^ hypotension (7 out of 11),^[8,10,12,14–17]^ and desaturation (3 out of 11).^[13,15,17]^ Amongst bradycardia patients, a total of 8 out of 9 cases returned to spontaneous circulation following initial treatment.^[8–10,14–17]^ Desaturation was observed in 3 out of 11 cases; mask ventilation, cardiopulmonary resuscitation, defibrillation, and reintubation were performed in some of these cases.^[13,15]^ In summary, cardiopulmonary collapse was reported in 8 out of 11 cases, wherein cardiopulmonary resuscitation was performed;^[8,10–15]^ however, 2 cases resulted in death.^[8,11]^ Case 1 reported by Bhavani et al^[8]^ returned to spontaneous circulation following resuscitation; however, the patient died on the 15th day after being transferred to the ICU. Mirza et al^[11]^ reported the death of another patient because of failure of resuscitation. In our case, the patient presented with bradycardia, hypotension, hypoxia, and weakness.

Different management practices were adopted according to clinical symptoms and signs. A total of nine cases were reported to have bradycardia. Therefore, atropine (4 out of 11)^[9,10,14,16]^ or glycopyrrolate (1 out of 11)^[11]^ were administered. Although the mechanism of anticholinergic agents remains unknown, and their effects are limited, the use of anticholinergic agents to treat s44ugammadex-induced bradycardia is still being considered in some case reports.^[18]^ However, some patients did not respond to anticholinergic agents such as atropine.^[16]^

In cases where hypotension was reported, vasopressors with epinephrine, norepinephrine, or ephedrine were administered.^[8,10–17]^ Additionally, other drugs, including lidocaine, calcium, and nicorandil, were administered to prevent further progression of cardiac ischemia and arrhythmia. Vasopressors have been preferred over anticholinergic agents for the treatment of sugammadex-induced bradycardia owing to the absence of known muscarinic effects of sugammadex^[19]^ and inadequate response to atropine.^[16]^ In our case, hypotension and bradycardia were effectively treated via administration of ephedrine and atropine.

The etiology of cardiac and pulmonary adverse effects in all the other cases is unknown. Two cases were suspected to be associated with hypersensitivity to sugammadex, as indicated by serum tryptase level and/or skin prick test.^[12,15]^ Another case was clinically diagnosed with hypersensitivity to sugammadex with tryptase and IgE levels within the normal range.^[17]^ These patients immediately experienced erythema, hypotension, or desaturation following the administration of sugammadex. Therefore, anaphylactic medications, such as methylprednisolone, and antihistamine drugs with hydroxyzine pamoate were administered.^[12,17]^

Pühringer et al^[18]^ reported that hypotension was observed in a study involving large doses of sugammadex. However, at three different doses of sugammadex (2, 4, and 16 mg/kg) in pooled phase I-III patients, the incidence of marked bradycardia was found to be 1%, 1%, and 5%, respectively.^[6]^ Upon reviewing these 11 cases, we found that the dose of sugammadex ranged from 2.08 to 4.17 mg/kg.^[8–17]^ In our case, the dosage of sugammadex used was 4.55 mg/kg. Therefore, the correlation between the dose of sugammadex and severity of bradycardia needs to be further investigated.

From 2014 to December 31, 2020, a total of 282 cases of major cardiac events were reported following sugammadex/sugammadex sodium/bridion administration as per the Food and Drug Administration *Adverse Event Reporting System* database. These events include bradycardia (n = 160), cardiac arrest (n = 110), cardiorespiratory arrest (n = 16), hypotension (n = 83), and decreased oxygen saturation (n = 55).^[20]^ However, in this study, we have investigated only 11 case reports from previous literature.^[8–17]^ Therefore, the incidence of adverse effects associated with sugammadex could have been underestimated.

## 4 Conclusions

Although sugammadex-induced bradycardia or cardiac arrest are rare, anesthesiologists should consider the possibility of the occurrence of such events. Immediate treatment with atropine and inotropic or vasopressors is recommended in such cases. Furthermore, advanced cardiac life support is required if initial management fails to manage the adverse effects.

## Author contributions

**Investigation:** I-Chia Teng, Yao-Tsung Lin, Zhi-Fu Wu.

**Supervision:** Chin-Chen Chu, Jen-Yin Chen.

**Writing – original draft:** I-Chia Teng, Zhi-Fu Wu.

**Writing – review & editing:** Ying-Jen Chang, Zhi-Fu Wu.
